# Supplementary material for: Stability of Manganese(II)–Pyrazine, –Quinoxaline or –Phenazine Complexes and Their Potential as Carbonate Sequestration Agents
Source: Molecules. 2022 Mar 2;27(5):1648. doi: 10.3390/molecules27051648 (PMC8911680; doi:10.3390/molecules27051648)
Supplement: Supplementary file 1 [file molecules-27-01648-s001.zip › molecules-1609949-supplementary.pdf]

molecules

MDPI

Supplementary Material

Stability of Manganese(II)–Pyrazine, –Quinoxaline or –Phenazine Complexes and Their Potential as Carbonate Sequestration Agents

José J. N. Segoviano-Garfias [1,*], Gabriela A. Zanor [1], Fidel Ávila-Ramos[1] and Egla Yareth Bivián-Castro [2]

[1] División de Ciencias de la Vida (DICIVA). Universidad de Guanajuato. Campus Irapuato-Salamanca. Ex Hacienda El Copal, Carretera Irapuato-Silao Km. 9, Irapuato, Gto. 36500 México.
[2] Centro Universitario de los Lagos. Universidad de Guadalajara. Enrique Díaz de León 1144, Col. Paseos de la Montaña 47460, Lagos de Moreno, Jalisco, México
* Correspondence: segovi@ugto.mx; Tel.: +524737405320

Supplementary Figures

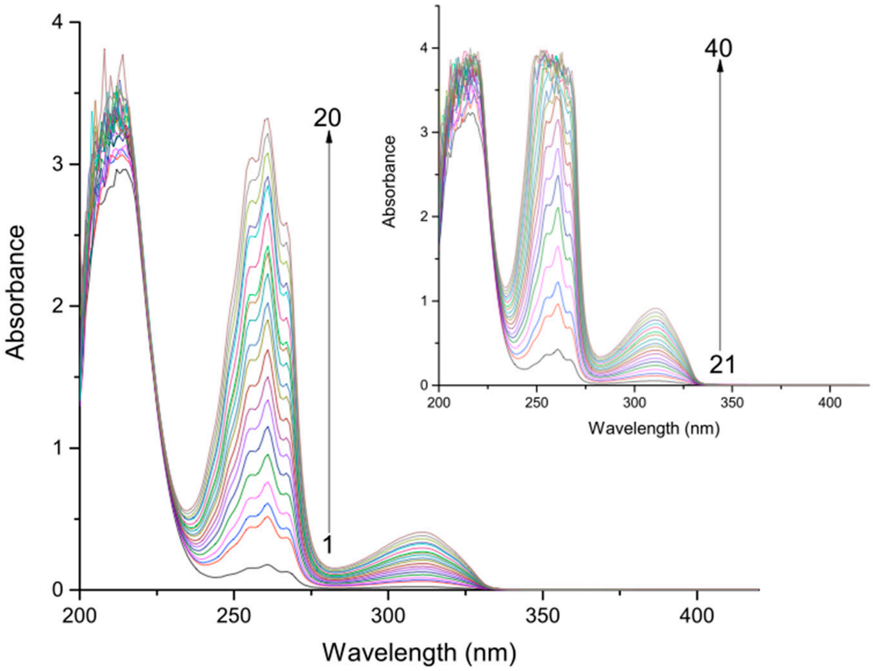

Figure S1. Absorption spectra of manganese(II)–pyrazine complexes in methanol solution. For spectra 1 to 14, [Mn(II)] = 348 μM and pyrazine concentration (μM): (1) 35; (2) 71; (3) 106; (4) 141; (5) 177; (6) 212; (7) 247; (8) 282; (9) 318; (10) 353; (11) 388; (12) 424; (13) 459; (14) 494; (15) 530; (16) 565; (17) 600; (18) 635; (19) 671; (20) 706. For spectra 21 to 40, [Mn(II)] = 696 μM and pyrazine concentration (μM): (21)71; (22) 141; (23) 212; (24) 282; (25) 353; (26) 424; (27) 494; (28) 565; (29) 635; (30) 706; (31) 777; (32) 847; (33) 918; (34) 988; (35) 1059; (36) 1130; (37) 1200; (38) 1271; (39) 1341; (40) 1412.

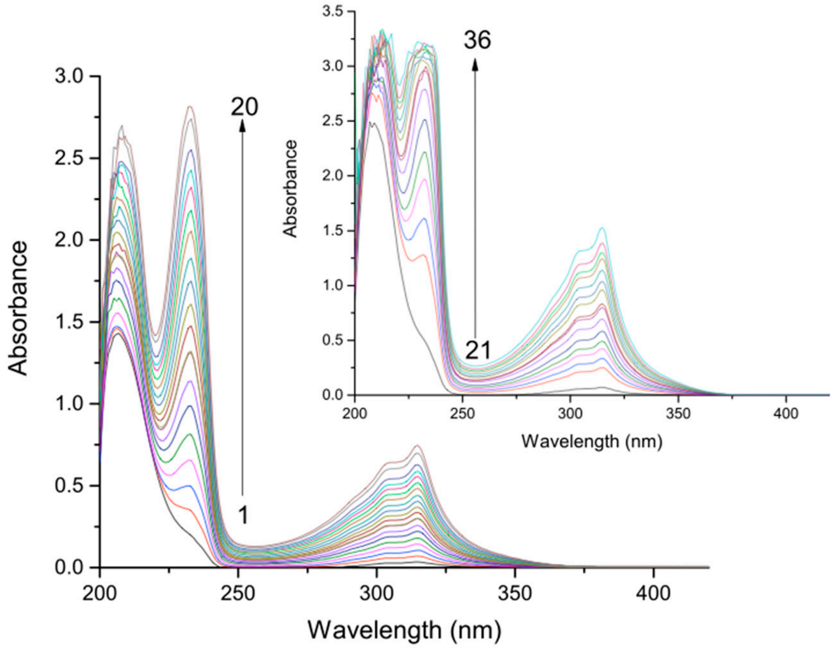

Figure S2. Absorption spectra of manganese(II)–quinoxaline complexes in methanol solution. For spectra 1 to 20, [Mn(II)] = 80 μM and quinoxaline concentration (μM): (1) 6; (2) 12; (3) 18; (4) 25; (5) 31; (6) 37; (7) 43; (8) 49; (9) 55; (10) 61; (11) 68; (12) 74; (13) 80; (14) 86; (15) 92; (16) 98; (17) 104; (18) 110; (19) 117; (20) 122. For spectra 21 to 40, [Mn(II)] = 160 μM and quinoxaline concentration (μM): (21)15.4; (22) 30.1; (23) 46; (24) 61.5; (25) 76.8; (26) 92.2; (27) 108; (28) 123; (29) 138; (30) 154; (31) 169; (32) 184; (33) 200; (34) 215; (35) 230; (36) 24.

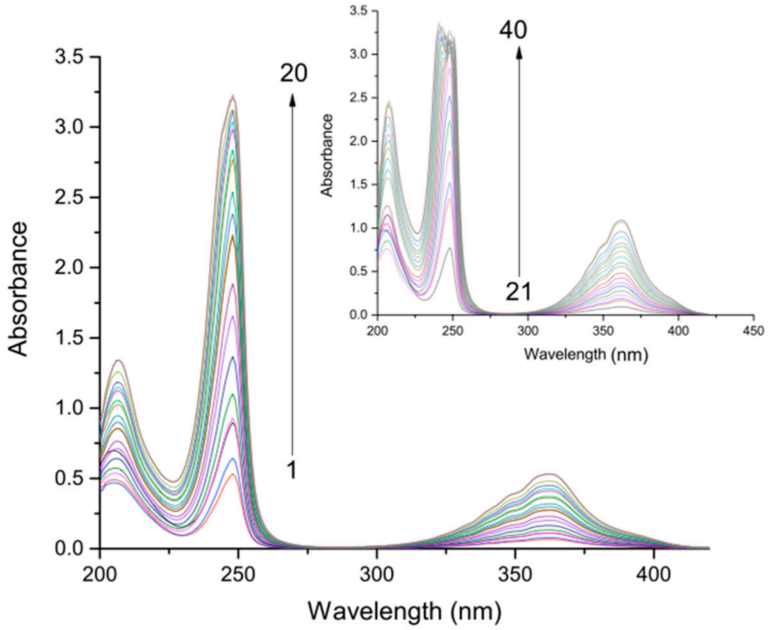

Figure S3. Absorption spectra of manganese(II)–phenazine complexes in methanol solution. For spectra 1 to 20, [Mn(II)] = 17.5 μM and phenazine concentration (μM): (1) 1.8; (2) 3.6; (3) 5.3; (4) 7.1; (5) 8.9; (6) 10.7; (7) 12.4; (8) 14.2; (9) 16.0; (10) 17.8; (11) 19.5; (12) 21.3; (13) 23.1; (14) 24.9; (15) 26.7; (16) 28.4; (17) 30.2; (18) 32.0; (19) 33.7; (20) 35.5. For spectra 21 to 40, [Mn(II)] = 35 μM and phenazine concentration (μM): (21) 3.6; (22) 7.1; (23) 10.7; (24) 14.2; (25) 17.8; (26) 21.3; (27) 24.9; (28) 28.4; (29) 32.0; (30) 35.5; (31) 39.1; (32) 42.6; (33) 46.2; (34) 49.7; (35) 53.3; (36) 56.8; (37) 60.4; (38) 63.9; (39) 67.5; (40) 71.0.

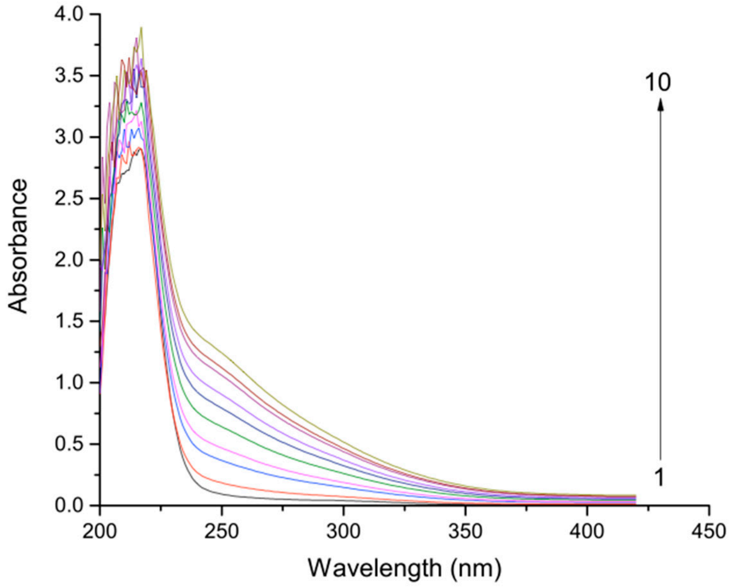

Figure S4. Absorption spectra of manganese(II)–carbonate complex in methanol solution. For spectra 1 to 10, [Mn(II)] = 335 μM and carbonate concentration (μM): (1) 66.9; (2) 134; (3) 201; (4) 268; (5) 335; (6) 402; (7) 469; (8) 536; (9) 603; (10) 669.

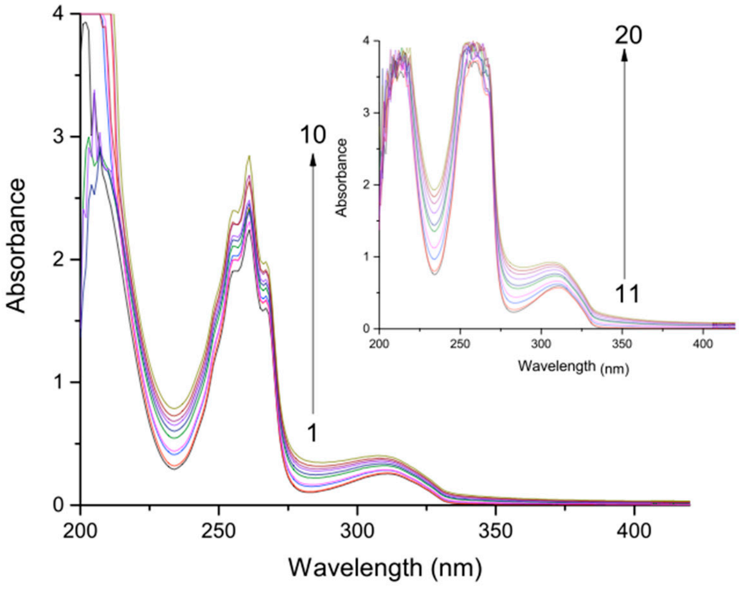

Figure S5. Absorption spectra of manganese(II)–pyrazine–carbonate complexes in methanol solution. For spectra 1 to 10, [Mn(II)] = 159 μM, [pz] = 349 μM and carbonate concentration (μM): (1) 30.2; (2) 60.4; (3) 90.6; (4) 120.8; (5) 150.9; (6) 181.1; (7) 211.3; (8) 241.5; (9) 271.7; (10) 301.9. For spectra 11 to 20, [Mn(II)] = 334 μM, [pz] = 700 μM and carbonate concentration (μM): (11) 66.9; (12) 133.9; (13) 200.8; (14) 267.8; (15) 334.7; (16) 401.6; (17) 468.6; (18) 535.5; (19) 602.5; (20) 669.4.

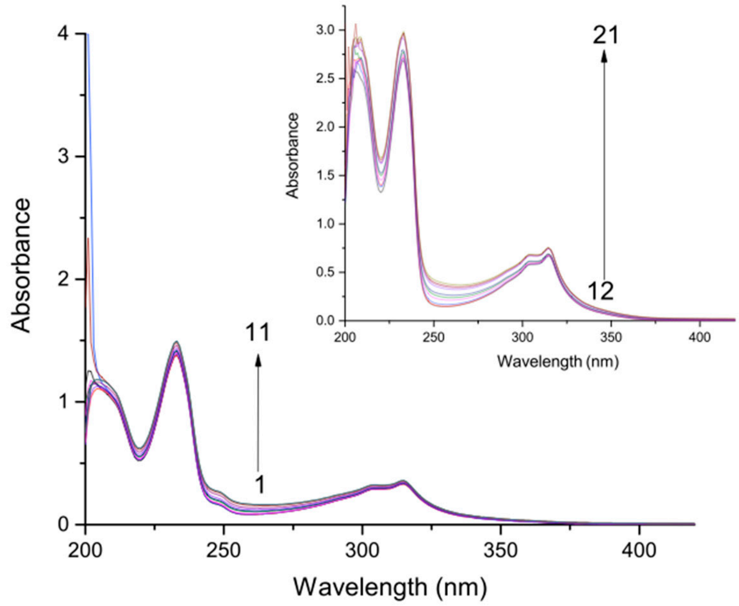

Figure S6. Absorption spectra of manganese(II)–quinoxaline–carbonate complexes in methanol solution. For spectra 1 to 11, [Mn(II)] = 31.8 μM, [qx] = 61.4μM and carbonate concentration (μM): (1) 7.5; (2) 15.1; (3) 22.6; (4) 30.2; (5) 37.7; (6) 45.2; (7) 52.8; (8) 60.3; (9) 67.9; (10) 75.4; (11) 83.0. For spectra 12 to 21, [Mn(II)] = 80 μM, [qx] = 123 μM and carbonate concentration (μM): (12) 16.0; (13) 32.0; (14) 48.0; (15) 64.0; (16) 80.0; (17) 96.0; (18) 112.0; (19) 128.0; (20) 144.0; (21) 160.0.

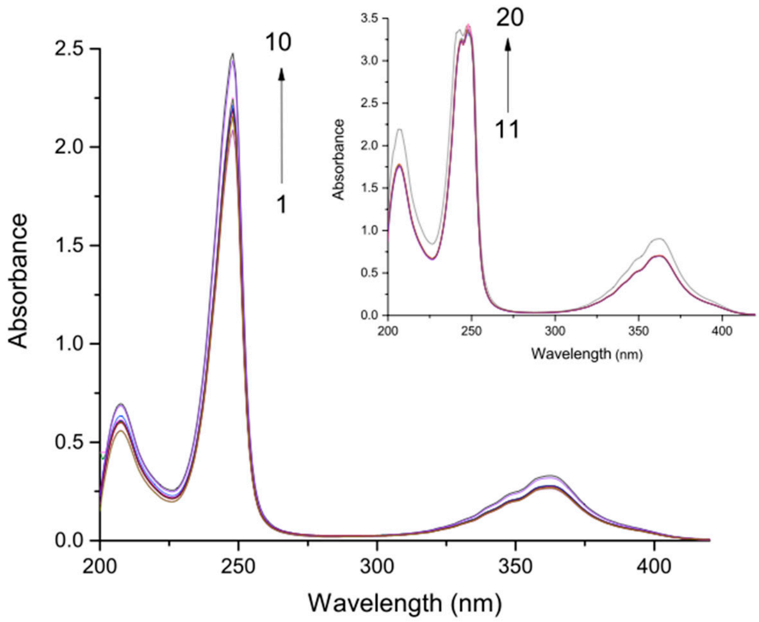

Figure S7. Absorption spectra of manganese(II)–phenazine–carbonate complexes in methanol solution. For spectra 1 to 10, [Mn(II)] = 9.6 μM, [fz] = 17.8 μM and carbonate concentration (μM): (1) 0.9; (2) 1.9; (3) 2.8; (4) 3.8; (5) 4.7; (6) 5.7; (7) 6.6; (8) 7.6; (9) 8.5; (10) 9.4. For spectra 11 to 20, [Mn(II)] = 17.6 μM, [fz] = 35.5 μM and carbonate concentration (μM): (11) 3.8; (12) 7.5; (13) 11.3; (14) 15.1; (15) 18.9; (16) 22.6; (17) 26.4; (18) 30.2; (19) 33.9; (20) 37.7.

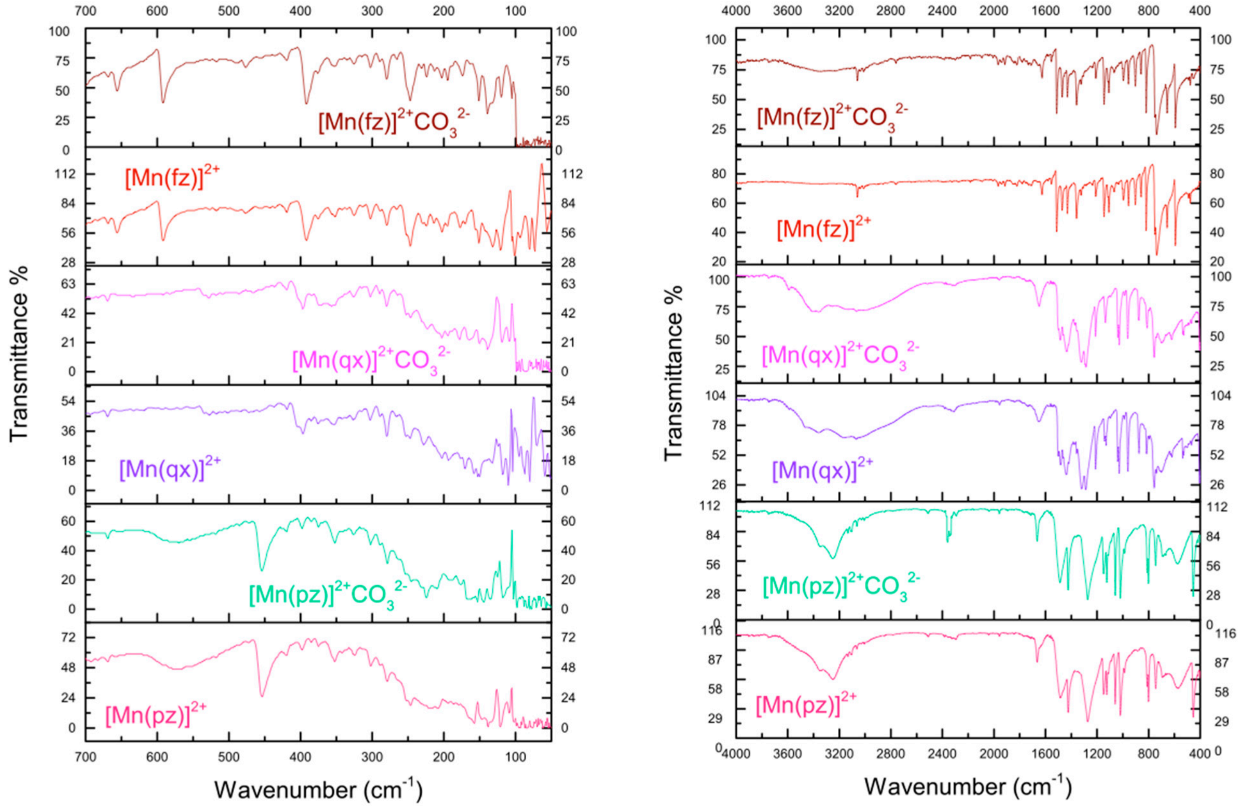

(**a**) (**b**)

Figure S8. (a) Far-infrared spectra of the different manganese(II) complexes obtained in this study: $[Mn(pz)]^{2+}$, $[Mn(qx)]^{2+}$, $[Mn(fz)]^{2+}$ and its carbonate complexes $[Mn(pz)]^{2+}CO_3^{2}$ $[Mn(qx)]^{2+}CO_3^{2-}$ and $[Mn(fz)]^{2+}CO_3^{2-}$. **(b).** Mid-infrared spectra of the different manganese(II) complexes obtained in this study: $[Mn(pz)]^{2+}$, $[Mn(qx)]^{2+}$, $[Mn(fz)]^{2+}$ and its carbonate complexes: $[Mn(pz)]^{2+}$ CO_3^{2-}, $[Mn(qx)]^{2+}CO_3^{2-}$, $[Mn(fz)]^{2+}CO_3^{2-}$.
